# Biological activities of fusarochromanone: a potent anti-cancer agent

**DOI:** 10.1186/1756-0500-7-601

**Published:** 2014-09-03

**Authors:** Elahe Mahdavian, Phillip Palyok, Steven Adelmund, Tara Williams-Hart, Brian D Furmanski, Yoon-Jee Kim, Ying Gu, Mansoureh Barzegar, Yang Wu, Kaustubh N Bhinge, Gopi K Kolluru, Quincy Quick, Yong-Yu Liu, Christopher G Kevil, Brian A Salvatore, Shile Huang, John L Clifford

**Affiliations:** Department of Chemistry and Physics, LSU-Shreveport, One University Place, Shreveport, LA 71115 USA; Department of Biological Science, LSU-Shreveport, Shreveport, USA; GlaxoSmithKline, Research Triangle, Park, NC USA; Department of Biochemistry and Molecular Biology, LSUHSC-Shreveport, Shreveport, USA; Department of Basic Pharmaceutical Sciences, University of Louisiana-Monroe, Monroe, USA; Department of Pathology, LSUHSC-Shreveport, Shreveport, USA; Department of Biological Sciences, Tennessee State University, Nashville, USA; US Army Institute of Surgical Research, Fort Sam, Houston, TX 78234 USA

**Keywords:** Pro-apoptotic, Anti-cancer, Anti-angiogenic, Small bioactive molecule, Fusarochromanone

## Abstract

**Background:**

Fusarochromanone (FC101) is a small molecule fungal metabolite with a host of interesting biological functions, including very potent anti-angiogenic and direct anti-cancer activity.

**Results:**

Herein, we report that FC101 exhibits very potent *in-vitro* growth inhibitory effects (IC_50_ ranging from 10nM-2.5 μM) against HaCat (pre-malignant skin), P9-WT (malignant skin), MCF-7 (low malignant breast), MDA-231 (malignant breast), SV-HUC (premalignant bladder), UM-UC14 (malignant bladder), and PC3 (malignant prostate) in a time-course and dose-dependent manner, with the UM-UC14 cells being the most sensitive. FC101 induces apoptosis and an increase in proportion of cells in the sub-G1 phase in both HaCat and P9-WT cell lines as evidenced by cell cycle profile analysis. In a mouse xenograft SCC tumor model, FC101 was well tolerated, non-toxic, and achieved a 30% reduction in tumor size at a dose of 8 mg/kg/day. FC101 is also a potent anti-angiogenenic agent. At nanomolar doses, FC101 inhibits the vascular endothelial growth factor-A (VEGF-A)-mediated proliferation of endothelial cells.

**Conclusions:**

Our data presented here indicates that FC101 is an excellent lead candidate for a small molecule anti-cancer agent that simultaneously affects angiogenesis signaling, cancer signal transduction, and apoptosis. Further understanding of the underlying FC101’s molecular mechanism may lead to the design of novel targeted and selective therapeutics, both of which are pursued targets in cancer drug discovery.

## Background

**Figure 1:**
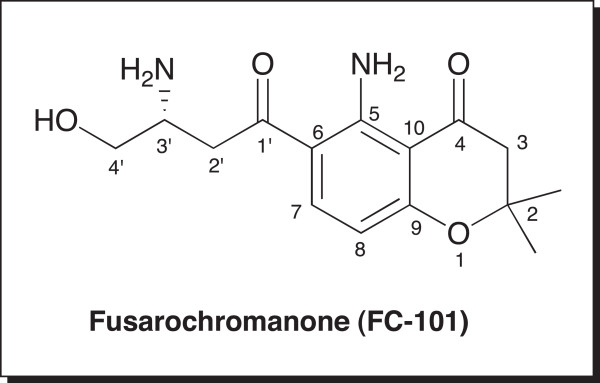
**Structure of fusarochromanone (FC101).**

Fusarochromanone (FC101) is a mycotoxin that is produced by the symbiotic fungus, *Fusarium equiseti,* found on decaying cereal plants from northern latitudes. FC101 was originally discovered to cause avian tibial dyschondroplasia (ATD) in broiler chickens [1, 2]. It also reduced hatchability in fertile eggs when birds were fed diets containing Fusarium-infected feed [1, 3]. This toxin is also suspected of being involved in etiopathogenesis of Kashin-Beck disease in children from northern China, Siberia, the former USSR, and Korea [4]. Lee *et al*. first isolated this molecule and determined its structure (without stereochemistry) via NMR and mass spectrometry [5]. FC101 is distinguished from other known chromanone natural products by the unique arrangement of the alternating *β*-keto-amine functionality along its upper face and two geminal methyl groups at C(2) (Figure 1). Using X-Ray crystallography, the absolute stereochemistry at C(3') was defined as 3’-*R*, according to the *Cahn-Inglold-Prelog* convention (Smith, unpublished observations). The amine group at C (3’) on the side-chain is in fact important to the molecule’s biological activity, since its acetylation drastically reduces the activity [6]. Wuthier reported that FC101’s inhibition of calcification in the legs of baby chickens was likely the result of its anti-angiogenic activity [7]. This effect is associated with a thickening of the cartilage on the tibial growth plate and a failure of this tissue to vascularize and calcify.

In addition to its anti-angiogenic properties in chickens, FC101 is also a potent anti-angiogenic agent in humans. FC101 has an IC_50_ value of 50 *nM* against human microvascular endothelial cells (NCI, unpublished). Those initial cell studies were done in the absence of any angiogenic factors.

It was later discovered that FC101 also acts directly on cancer cells. A 60-cell-line drug screening assay performed by the National Cancer Institute (NCI) revealed that FC101 inhibited the proliferation of 35 of 58 human cancer cell lines with IC_50_’s of less than 100 nM [NCI, unpublished]. The most sensitive cell lines were human melanoma, small cell lung carcinoma, and colon adenocarcinoma, with IC_50_ values all below 10 nM. The NCI also conducted a COMPARE screen on FC101. This screen applies algorithms that are used to assess the mode of action of a test compound. In this screen, FC101 inhibition was studied in the same 60 cell-line drug screening assay as mentioned above. These patterns were then compared to those found in a library of over 50,000 compounds. The data generated from the COMPARE test is represented as a Pearson correlation coefficient. Correlations greater the 0.8 indicate that the test molecule inhibits cellular growth in a similar manner to a compound found in the NCI database. Correlations below 0.6 are thought to have minimal, if not altogether different modes of action. The NCI COMPARE correlation factor for FC101 was 0.475, indicating that FC101 is unique in its mode of action [NCI, unpublished].

Another unique attribute of FC101 is its intrinsic fluorescence, with a maximum excitation at 385 nm and emission at 457 nm. A recent study utilized this intrinsic fluorescence to investigate the kinetics and uptake of FC101 by tumorigenic cells *vs.* normal cells in a rodent model analyzed by confocal microscopy [8]. This study reported an increased uptake of FC101 and growth inhibition in tumorgenic B16 melanoma and MCF-7 breast adenocarcinoma cells, as compared to the normal cardiac fibroblast cells. This group also reported experimental and *in-silico* values for a series of physiochemical properties (LogP, LogD, polar surface area, hydrogen bonding, molecular flexibility) that contribute to the bioavailability of FC101. They concluded that FC101 shows very good cell permeability and intestinal absorption, meeting the criteria for therapeutic drugs that were established by Lipinski *et al.* [9–11].

In this study we also tested the hypothesis that FC101’s molecular targets (i.e., interacting biomolecules) could be regulators of angiogenesis, cancer signal transduction, cell proliferation, and/or programmed cell death (apoptosis). The detailed effects of FC101 on these processes were determined for a panel of human tumor cell types (skin squamous cell carcinoma [SCC], breast, prostate, and bladder). In addition, we tested the tumor suppressive effect of FC101 *in vivo*, in a mouse tumorigenicity model, against human skin SCC cells. SCC is the most aggressive form of non-melanoma skin cancer.

## Results

**Figure 2:**
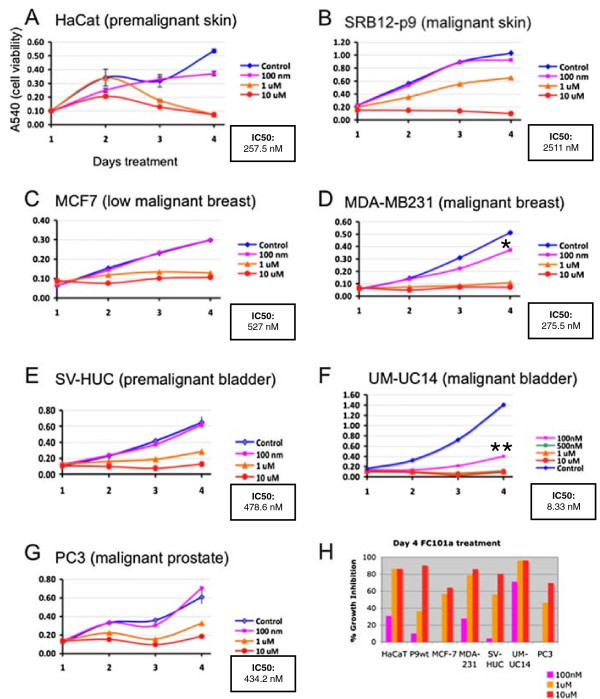
**Comparison of the dose-response and time-response impact of FC101 treatment on cell viability of seven cell lines. A-G**: A540 is the absorbance at 540 nm, which positively correlates with cell number. Error bars are shown for triplicate cultures. The graphs are representative of at least three experiments having similar results. **H**: The percent growth inhibition at day 4, calculated as (A540C-A540T/A540)x100, where A540C is the control value and A540T is the treated value. The graph lines in A-G are color matched to the bars in **H**.

It was previously reported in a mouse xenograft model for melanoma that FC101 induced expression of active caspase-3 [12]. Furthermore, our preliminary studies in yeast have shown that Yca1 is involved in the response to FC101-treatment (Williams-Hart, unpublished). Yca1 is a caspase-like protein that has been shown to be important for executing apoptotic-like responses in yeast [13]. This finding led to the hypothesis that the tumor suppressive effects of FC101 may result from changes in the regulation of apoptotic signaling. In order to determine whether FC101 can induce cell death in culture, a preliminary indicator of apoptosis, several cell lines derived from different human tumor types were treated with a range of doses of FC101 over a course of four days. The MTT cell viability assay was used to obtain an indirect measure of cell number. All tumor cell types were sensitive to FC101 in a dose dependent manner, and in the case of the breast and bladder cancer cell lines, the more malignant cells were more sensitive to FC101 than the less malignant ones (Figure 2, compare 100 nM curves in panel C to D, and E to F).

These results are more clearly indicated in Figure 2H, where the percent growth inhibition for all 7 cell lines is shown for the day 4 time point (color of lines in graphs in Figure 2A-G corresponds to color of bars in Figure 2H). The UM-UC14 cells were the most sensitive of all of the cell lines; the 100nM dose drastically diminished cell number compared to other cells lines. It should be noted that this was the fastest growing cell line of the group tested (Figure 2F, compare the y-axis scale to the other graphs).

Since the MTT assay only measures cell metabolism/mitochondrial respiration, which is an indirect measure of cell number, it cannot readily determine whether reduced cell number is caused by suppression of proliferation, or the induction of apoptosis. In order to determine which process FC101 predominantly affects, we measured its effect on the cell cycle distribution. The HaCat premalignant keratinocyte and P9 WT skin SCC cell lines were chosen for this analysis, because these cell lines have been extensively studied in this context with other apoptosis-inducing drugs.

**Figure 3:**
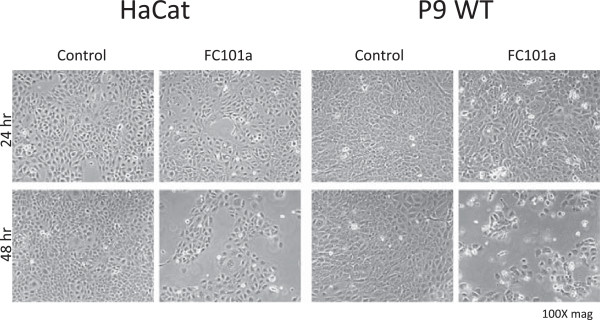
**Cells were plated at a density of 1.5 × 10** ^**6**^ **cells/10 cm plate and treated the following day with 10 μM FC101.** Cells were photographed on a phase contrast microscope at 100X magnification.

**Figure 4:**
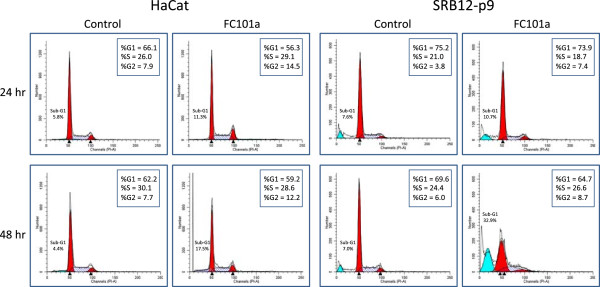
**Differential effect of FC101 on cell cycle distribution and apoptosis induction in HaCaT and SRB12-p9 cells.** Cells were plated and treated as in described in Figure 3 and harvested for FACS analysis. Y-axis indicates cell number, X-axis indicates intensity of propidium iodide staining. The numbers in boxes indicate the percentages of live cells in each phase of the cell cycle. The percent of total particles counted that are sub-G1 in size are also indicated.

Cells were treated for 24 and 48 hours with FC101 (10 μM). These time points were chosen because marked effects of FC101 on viability were first detected after 48 hours of treatment (Figure 2A and B, compare 1 and 2 day time points). As with the MTT results for both cell lines, there was little effect of FC101 treatment after 24 hours (Figure 3, upper row, FC101 panels). However, after 48 hours there were fewer attached cells in both cell lines, with a stronger effect visible for the P9 WT cells (Figure 3, lower panels). The presence of many rounded cells detaching from the plate is an indicator of cell death, possibly by apoptosis. In order to verify whether FC101 did indeed induce apoptosis, and whether there were any changes in the proliferative behavior of the cells, FACS analysis was performed for the same dose and time points of treatment. Treatment with FC101 for 24 hours caused an increase in percentage of cells in the 2 N DNA content peak, indicating cells in the G2 and M phases of the cell cycle for both cell lines (Figure 4, compare FC101 panels to controls in top row). The G2 phase is the point at which the DNA has been fully replicated, but prior to mitosis. This effect was accompanied by a modest increase in the percentage of particles that were smaller in size than G1 cells (the sub-G1 fraction) for both cell lines, indicating a modest increase in apoptosis with treatment. An enhanced fraction of cells in the 2 N peak was still observed after 48 hours of treatment, but the sub-G1 fraction was markedly increased compared to controls (Figure 4, lower panels). The increase in the percentage of cells in the 2 N peak was greater at both time points for the HaCaT cells compared to SRB12-p9 cells. However, the induction of apoptosis, as measured by the size of the sub-G1 fraction, was greater for the SRB12-p9 cells.

Programmed cell death can proceed through either caspase-dependent or independent routes [14]. To determine the mechanism of FC101-induction of apoptosis, MDA-MB-231 breast cancer cells were exposed to FC101 (0–1 μM) for 24 h, followed by western blotting analysis to probe the differential expression and activities of the pro-apoptotic, anti-apoptotic, and caspase proteins involved in apoptosis.

**Figure 5:**
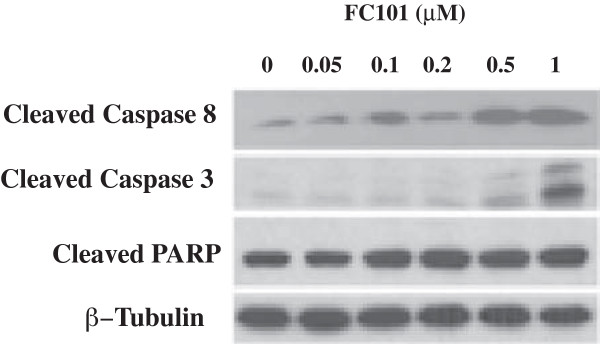
**MDA-MB-231 cells, grown in 6-well plates, were treated with FC101 (0–1 μM) for 24 h, followed by western blotting with antibodies to cleaved caspase-3, cleaved caspase 8, and cleaved PARP.** Beta-tubulin was used for loading control. Immunoblots shown are representative of three independent experiments that showed similar results.

**Figure 6:**
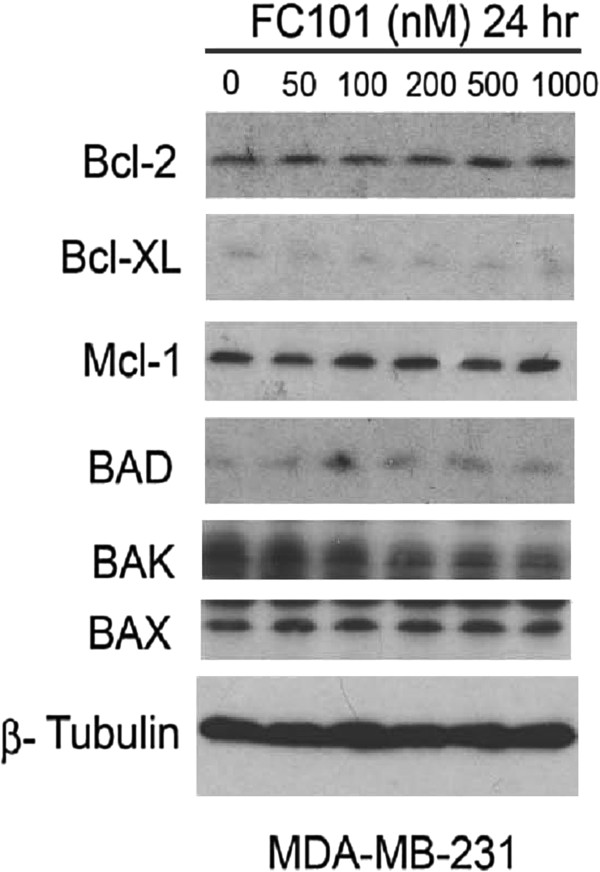
**MDA-MB-231 cells, grown in 6-well plates, were treated with FC101 (0–1 μM) for 24 h, followed by western blotting with antibodies to anti-apoptotic proteins (Bcl-2, Bcl-XL, Mcl-1) and pro-apoptotic proteins (BAD, BAK, BAX).** Beta-tubulin was used for loading control. Immunoblots shown are representative of three independent experiments that showed similar results.

FC101 induces the cleavage of both caspase-3 and PARP, a well-known substrate for activated caspases (Figure 5). This indicates that FC101 activates the caspase signaling cascade. However, FC101 does not affect the expression of the anti-apoptotic proteins, Bcl-2, Bcl-XL, Mcl-1, or the pro-apoptotic proteins BAD, BAK, BAX (Figure 6). Thus, although FC101 activates apoptosis in a caspase-dependent manner, this activity does not involve a mitochondrial-mediated intrinsic mechanism. Furthermore, treatment with FC101 increases the activity of caspase-8, thus substantiating that FC101 initiates apoptotic cascades through a death receptor mediated extrinsic mechanism involving caspase-8 activation (Figure 5).

**Figure 7:**
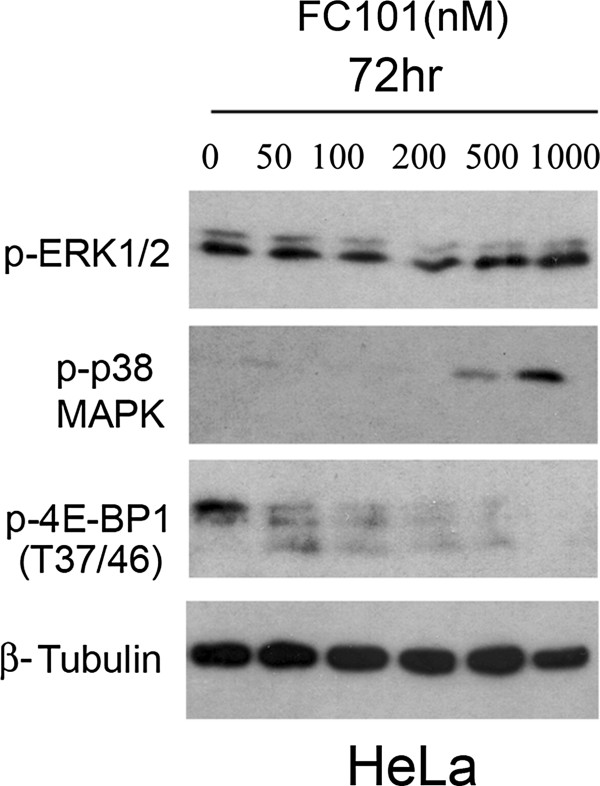
**HeLa cells, grown in 6-well plates, were treated with FC101 (0–1 μM) for 72 h, followed by western blotting with antibodies for p-4E-BP1 (Thr37/Thr46) phospho-Erk1/2 (Thr202/Tyr204), p38, phospho-p38 (Thr180/Tyr182).** Beta-tubulin was used for loading control. Immunoblots shown are representative of three independent experiments that showed similar results.

Mitogen Activated Protein Kinases (MAPKs) are also involved in programmed cell death [15] and are frequently implicated in apoptotic responses by small molecule chemotherapeutic agents. Next, we determined whether FC101-induced cell death involves MAPKs, namely activation of Erk1/2 and p38 downstream pathways. For this, HeLa cells were exposed to FC101 (0–1 μM) for 48 hours, followed by immunoblot analysis. As shown in Figure 7, FC101 did not affect phosphorylation of ERK1/2, but induced phosphorylation of p38 in a concentration-dependent manner. Note: p-ERK1/2 level is often correlated to increased cell survival, whereas phosphorylated p38 level is frequently associated with stress response or cell death. This could suggest that FC101 induction of cancer cell death is in part due to the activation of the p38 MAPK signaling pathway.

The mTOR signaling pathway also plays a central role in the regulation of cell proliferation and growth [16]. Inhibition of mTOR by rapamycin downregulates cyclin D1 expression [17] and Rb phosphorylation [18], resulting in cell cycle arrest during the G_1_-G_0_ phase. Since we found that FC101 arrests cells in G_1_-G_0_ phase of the cell cycle, we hypothesized that the cell-cycle modulatory effect of FC101 is linked to inhibition of the mTOR pathway. To test this hypothesis, we examined the effects of FC101 on mTOR signaling in Hela cells. As shown in Figure 7, FC101 lowers the levels of p-4E-BP1 (T37/46), one of the best-characterized downstream effector molecules of mTOR [16] in a concentration-dependent manner.

It is clear that the anti-proliferative and pro-apoptotic effects of FC101 are mediated through both the activation of p38-MAPK and the inhibition of mTOR signaling pathways. Further investigation of the underlying molecular mechanisms may lead to the design of other novel small molecules targeting both the mTOR and MAPK pathways.

**Figure 8:**
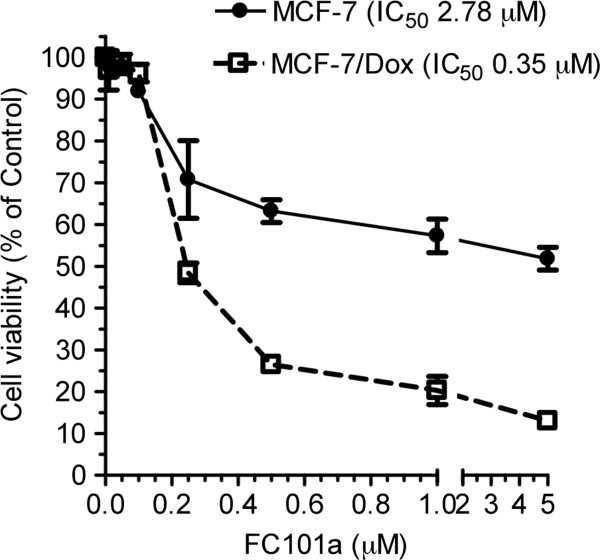
**The growth inhibitory effect of FC101 on the MCF-7 and MDR MCF-7/Dox cells.**

We also investigated the effects of FC101 on multi-drug resistant (MDR) cells, MCF-7/Dox. We found that FC101 has a more pronounced anti-cancer effect (~8-fold lower IC_50_) on MCF-7/Dox than on their parental cell line, MCF-7 (Figure 8). The MCF-7/Dox cells over-express two major genes responsible for drug resistance, one of which is glycosylceramide synthase (GCS) [19]. The fact that FC101 displays greater potency towards MDR cells suggests that it should be further explored as a sensitizing agent that may offer a therapeutic advantage. We are also currently designing experiments to more fully elucidate the detailed mechanism underlying this favorable feature of FC101.

**Figure 9:**
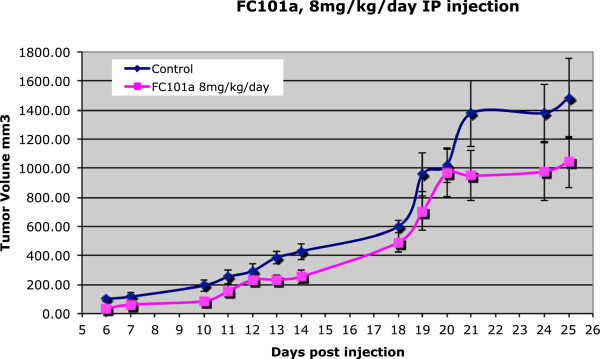
**Immunocompromised mice (SCID) were injected subcutaneously with 1x10** ^**6**^ **SRB12-p9 cells on day 0, and injected intraperitoneally with 8 mg/kg/day FC101 dissolved in PBS, or PBS alone as a control.** The tumor volumes were determined by caliper measurement on the days indicated.

Like most other bioactive natural flavonoids, FC101 has modest *in-vivo* activity. In a mouse xenograft skin SCC tumor model, FC101 was well tolerated and non-toxic, but it required a dose of 8 mg/kg/day treatment to achieve a 30% reduction in tumor size, compared to untreated controls (Figure 9, compare pink line with blue line).

**Figure 10:**
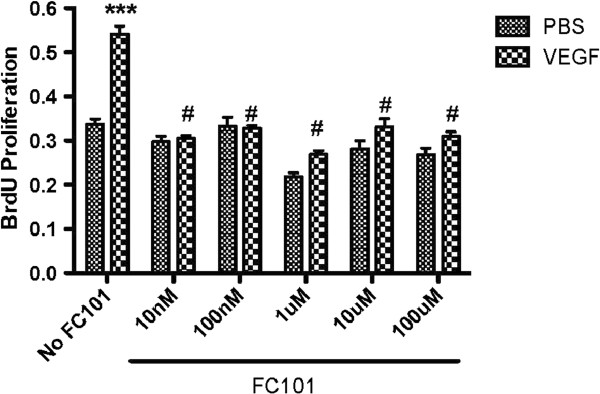
**Suppression of VEGF-induced proliferation of mouse microvascular endothelial cells (MS1).**

To assess the direct anti-angiogenic properties of FC101, we used the MS1 mouse microvascular endothelial cell line, which was selected for its high VEGFR2 expression and responsiveness to VEGF functions [20]. Thus, these cells are a good *in-vitro* proxy for optimal VEGF dependent responses that would be seen *in vivo*. FC101 displayed potent growth inhibition of MS1 cells with an IC_50_ < 50 nM after 4 hours treatment. The dose-dependent effect of FC101 on VEGF-stimulated MS1 cell growth was also examined using an ELISA-based BrdU incorporation assay. We determined that FC101 significantly inhibits VEGF-dependent endothelial cell proliferation at all doses beginning at 10 nM (Figure 10).

## Discussion

FC101 is a small molecule fungal metabolite that has very potent anti-angiogenic activity and direct anti-cancer activity, and it exhibits potent *in-vitro* growth inhibitory effects (IC50 < 2.5 μM for all cell lines tested). However, the precise biological target of FC101 remains unknown.

The data show that FC101 has the potential to be an effective anti-cancer therapeutic drug, because it suppresses both angiogenesis and tumorigenesis. *In-vitro* and *in-vivo* observations both indicate that FC101 is a powerful cytotoxic agent with strong selectivity towards cancer cells versus normal cells. Its broad-spectrum inhibitory effect on cancer cell lines and its direct inhibition of endothelial cell growth suggest that it could be beneficial for treating a variety of human cancers. *In-vitro* experiments showed significant differential sensitivity in a variety of malignant cell types (*e.g.,* melanoma) vs. pig-1 melanocytes, immortalized non-malignant skin cells that serve as a control for human melanoma cell lines^8^. Cellular proliferation was measured by 3H-thymidine incorporation (cpm/well). FC101 inhibited greater than 90% of incorporation with as little as 10 nM in melanoma cells. In contrast, normal melanocytes were significantly less sensitive to FC101 (*ca.* 100-fold; data are not shown). In fact, FC101 dosages of 0.1 and 1 ng/mL caused stimulation of melanocyte proliferation; inhibition occurred only after exposure to high levels of FC101 for prolonged period of time, 8–12 days. An *in vivo* mouse xenograft SCC tumor model has also shown that FC101 is well tolerated and non-toxic to normal animal tissues.

Another unique attribute of FC101 was its differential growth inhibitory effect towards chemotherapy-resistant cancer cells. FC101 displayed a very potent sub-micromolar IC_50_ value against the MDR cells, MCF-7/Dox, which overexpress the enzyme, GCS. GCS converts ceramide to glucosylceramide, thereby deactivating it. Ceramide is an important lipid second messenger that mediates growth arrest and apoptosis of cells. Ceramide-induced apoptosis contributes to the therapeutic efficiencies of anthracyclines, taxanes, cytokines, and irradiation, and the over-expression of GCS has been identified as a possible mechanism for resistance to chemotherapy in a number of human cancers [19]. Indeed, GCS overexpression is displayed in MDR cell lines of breast, ovarian, cervical, and colorectal cancers [16]. Furthermore, these same investigators have demonstrated that inhibition of GCS sensitizes MDR cells to anticancer drugs [19, 21]. Inhibition of GCS with small molecules, such as D-*threo*-1-phenyl-2-decanoylamino-3-morpholino-1–propanol (PDMP), sensitizes cancer cells to doxorubicin, paclitaxel and vincristine [22]. The suppression of GCS expression by MBO-asGCS restores ceramide signaling (particular C_18_-ceramide) during the course of doxorubicin treatment, thereby promoting caspase-executed apoptotic death in cells. Thus, targeting GCS would promote caspase-executed apoptotic signaling mediated by ceramide, thereby eliminating tumors with poor response to conventional chemotherapy, such as doxorubicin. However, a therapeutic agent that efficiently inhibits GCS *in vivo* remains to be developed. FC101 is a promising candidate for such an agent. Our future studies will address whether FC101 suppresses GCS expression or activity to reverses drug resistance.

Figure 4 illustrates that FC101 has at least one other beneficial biological effect besides apoptosis induction. The increased fraction of cells in the 2 N DNA content peak G2 and M phases of the cell cycle suggests that FC101 could slow the rate of cell proliferation, possibly through an effect on the transition from G2 to mitosis. Further experiments, such as detailed analysis of cell cycle protein levels and phosphorylation states as well as cytological observation of treated cells using DNA staining, will be necessary to distinguish between cells in G2 and M phase.

We have shown that FC101’s molecular mode of action involves three key cellular mechanisms including, extrinsic apoptosis, MAPK, and mTOR signaling pathways. FC101 activates caspase-3, which initiates degradation of the cell in the final stages of apoptosis. Increased PARP cleavage confirms the activation of the caspase cascade. FC101 also activates caspase-8, as evidenced by increased caspase-8 cleavage, further substantiating that FC101 induces apoptosis through an extrinsic pathway.

FC101 does not affect the expression of the anti-apoptotic proteins, Bcl-2, Bcl-XL, Mcl-1, or the pro-apoptotic proteins BAD, BAK, BAX. This indicates that FC101’s induction of apoptosis does not occur through an intrinsic mechanism. The data support the notion that FC101 induces caspase-dependent apoptosis, which implies that the extrinsic pathway is involved in this process. Future experiments will explore the effects of FC101 on the expression of death-receptor signaling proteins (TNF-α and FAS) to further confirm that FC101-induction of apoptosis is death receptor mediated through an extrinsic pathway.

FC101 did not affect phosphrorylation of ERK1/2, but it induced phosphorylation of p38 MAPK in a concentration-dependent manner (HeLa cells, 0–1 μM). Note p-ERK1/2 levels are often correlated to increased cell survival, whereas p38 levels are frequently associated with stress response or cell death. In addition, FC101 inhibits the phosphorylation of 4E-BP1 (T37/46), a substrate of mTOR. Since mTOR is a master kinase that controls cell growth and survival, this suggests that FC101 may induce cell death by simultaneously activating p38 MAPK and inhibiting mTOR signaling.

We have found that the cell-permeable small molecule, FC101, affects a diverse group of biological targets involved in cellular signal transduction. Collectively, our findings suggest that FC101 induces a series of caspase-dependent events in the extrinsic cascade, resulting in apoptosis, and we have shown that FC101 simultaneously affects two downstream protein substrates involved in two distinct kinase signaling pathways (mTOR and MAPK signaling pathways).

We believe that FC101 holds promise as an anticancer agent due to its unique molecular mechanism especially as a modulator of both mTOR and MAPK, two master kinases that regulate cell proliferation and growth [16]. Further elucidation of the underlying molecular mechanism may lead to the design of a new class of targeted cancer therapeutics.

Our data also shows that FC101 inhibits VEGF-dependent proliferation of microvascular endothelial cells (MS1 cells), indicating a strong anti-angiogenic effect. As shown in Figure 10, all doses of FC101 significantly inhibited VEGF-mediated Brdu incorporation. One limitation of prior work is that it was performed in the absence of any endothelial cell growth factors. This made it unclear how effective FC101 would be in conjunction with a growth factor like VEGF.

FC101 has shown high potency *in vitro*, coupled with low toxicity toward normal tissues and little or no adverse effects in various animal studies. In one report on the use of FC101 in a melanoma xenograft model in mice, Dréau *et al*. commented on the potent cytotoxicity of FC101, but also noted only minimal anti-angiogenic activity, despite the strong activation of caspase-3 at the periphery [12]. This model also confirmed other previous reports that FC101 is less potent *in-vivo* than *in-vitro*. Identifying the pharmacokinetic parameters of biologically active compounds is an essential step in the development of potential drug candidates. Typical pharmacokinetics parameters are the *in vivo* absorption, distribution, metabolism, and elimination of compounds in blood and tissues over time. FC101 is a flavonoid, and over 4,000 natural compounds have been characterized as such. These molecules are usually poorly to moderately water-soluble. They are also rapidly metabolized and degraded *in vivo*. Flavonoids generally bind tightly to serum proteins (*e.g.,* serum albumin) and thus substantial amounts are inaccessible to the desired biological targets.

Through a previous study in Dr. Wuthier’s lab (one of our collaborators), we have seen that FC101 does indeed bind to BSA with a binding constant of 2.47 ± 0.17 x 10^8.^ To achieve efficacy *in vivo*, promising preclinical drug candidates like FC101 need to exploit pharmacologic delivery routes that facilitate sustained release of the active form of the drug at the desired site of action. Incorporation of flavonoids into lipid- or polymer-based nanoparticles appears promising. This markedly helps drug delivery, as these nanoparticles can protect the drug from degradation in the gastrointestinal tract and degradation from first-pass metabolism in the liver, by virtue of the unique absorption mechanism of these conjugates through the lymphatic system.

Thus, although we view FC101 as a promising drug candidate for structure-based lead optimization, its stability, bioavailability, and *in vivo* distribution appear to be sub-optimal. Currently, we are synthesizing new FC101 analogs with greater *in-vivo* potency and also attempting to identify the precise biological target(s) of FC101.

## Conclusion

Scientists have traditionally turned to nature to find new lead compounds for fighting disease. Fungus and molds have produced many important new lead compounds for drug development. That is how FC101 was discovered, and it represents an important new lead compound for the treatment of cancer. However, the precise biological targets of FC101 remain unknown. Thanks to advances in computer-based assays and chemical synthesis, there are new ways to predict a drug’s mechanism of action and to synthesize more biologically active analogs of promising lead compounds. The chemical synthesis of novel analogs, cell-based as well as protein-based assays that probe specific signaling pathways will play a significant role in our future development of FC101 for cancer therapy.

## Methods

### Source of drug

FC101 in its free base form (MW 292.3) was isolated and purified from *Fusarium equiseti* (originally supplied by Dr. Xie at the University of Minnesota). This fungus is found exclusively in the arctic latitudes and grown on rice cultures by Dr. Brian Furmanski. FC101 was then crystallized in the presence of phosphoric acid to form the more stable phosphate salt form (MW 390.3). Its purity (>98%) was confirmed by ^1^H-NMR, ^13^C-NMR, and UV–vis spectroscopy.

### Cell lines and culture

Seven human tumor cell lines were used in this study. HaCat (pre-malignant skin, a gift from Prof. N.E. Fusenig, Deutsches Krebforschungszentrum), SRB12-p9 (malignant skin SCC, a gift from Dr. Reuben Lotan, Univ. of Texas-MD Anderson Cancer Center), MCF-7 (low malignant mammary gland adenocarcinoma), MDA-231 (malignant mammary gland adenocarcinoma), SV-HUC (premalignant bladder), UM-UC14 (malignant bladder, a gift from Dr. H.B. Grossman, Univ. of Texas-MD Anderson Cancer Center), and PC3 (malignant, prostate). MCF-7, MDA-231, SV-HUC and PC3 cells were obtained from the American Type Culture Collection (Manassas, VA). HaCaT cells were cultured in 4x modified Eagle’s medium (MEM) media (1.4 mM Ca^2+^), supplemented with 5% fetal calf serum (FCS) and 1% Penicillin Streptomycin Solution (Pen-Strep). SRB12-p9, MCF-7, MDA-MB231, and PC3 cells were cultured in Dulbecco’s MEM (DMEM)/F12 medium containing 10% FCS and 1% Pen-Strep. UM-UC14 cells were cultured in 50% DMEM/F12 media and 50% DMEM low glucose media containing 10% FCS and 1% Pen-Strep. SV-HUC cells were cultured in HyQ Ham’s/F-12 media with 7% FCS and 1% Pen-Strep. For passaging, subconfluent cells were incubated with 0.1% trypsin/2 mM EDTA and suspended in media before replating. All cells were cultured at 37°C in the humidified atmosphere of 5% CO_2_/95% air.

### MTT assays

Cell viability was determined by the MTT 3-(4, 5-dimethyltiazol 2-yl)-2,5-diphenyltetraolium bromide assay. Briefly, cells were plated in triplicate wells at varying densities (1,000 cells/well for HaCaT, SRB12-p9, MCF7, MDA-MB231; 1,500 cells/well for MCF7, PC3; 2,000 cells/well for SV-HUC) in 100 μl growth media in 96-well plates and treated with the FC101 at the concentrations indicated in Figure 4. After a range of treatment times a solution of MTT (20 μl of a 12 mM solution in PBS) was added and incubated for 2 hours at 37°C. The cells were washed gently with PBS, and 100 μl of DMSO was then added to the wells followed by mild shaking to dissolve the MTT precipitate. Absorbance at a wavelength of 540 nm was measured for each well using a Wallac Victor3 1420 Multilabel multi-well plate reader (Perkin-Elmer).

### Cell viability assays for multi-drug resistant MCF-7/Dox cells

Cell viability was analyzed by quantitation of ATP, an indicator of active cells, using the CellTiter-Glo luminescent cell viability assay (Promega, Madison, WI). Briefly, cells (4,000 cells/well) were grown in 96-well plates with 10% FBS RPMI-1640 medium for 24 hr. FC101 was introduced into cells by Lipofectamine 2000 (vehicle control) in Opti-MEM I reduced-serum medium, for a 4 hr incubation. Cells were then incubated with increasing concentrations of FC101 in 5% FBS medium for an additional 72 hr. Cell viability was determined by the measurement of luminescent ATP in a Synergy HT microplate reader, following incubation with CellTiter-Glo reagent. Triplicate experiments were repeated two times.

### Cell cycle analysis

The cell cycle profile was determined by cell cycle flow cytometry based on cellular DNA content, using an Epics Profile II cell sorter (Coulter Electronics, Inc.). Cells were treated with FC101 or DMSO vehicle control for the indicated durations, trypsinized, collected and pelleted together with any material floating in the medium. Cells were fixed in cold 70% ethanol, resuspended in PBS at a density of > 10^6^ cells/ml followed by RNase A (1 mg/ml) treatment, addition of propidium iodide (20 μg/ml final concentration) and analysis by flow cytometry. The percentage of cells in different phases of the cell cycle were determined from the raw data using the *ModFit V3.2.1* flow cytometry software. Apoptosis was assessed by determining the amount of sub-G1 sized particles.

### Western blot analysis

Western blotting was carried out as described previously [14]. Briefly, control and treated cells (0–10 μM) were washed twice with cold PBS and then lysed in the lysis buffer [50 mmol/L Tris, pH 7.2; 150 mmol/L NaCl; 1% sodium deoxycholate; 0.1% SDS; 1% Triton X-100; 10 mM NaF, 1 mM Na_3_VO_4_; Protease inhibitor cocktail (1:1000 dilution, Sigma). Lysates were sonicated for 10 seconds and centrifuged at 13,000 X g for 10 minutes at 4°C. Protein concentration was determined using the BCA assay (Protein Assay Kit, Pierce). Protein samples (~40 μg) were electrophoresed on 8–12% SDS-PAGE, transferred to PVDF membrane (Millipore, USA), and probed with respective primary and secondary antibodies. The following primary antibodies were used: p38, phospho-p38 (Thr180/Tyr182), PARP, Bcl-2, Mcl-1, surviving, (all from Santa Cruz Biotechnology), Bcl-xL, BAK, BAX (Biomedia), p-4E-BP1(Thr37/Thr46), caspase 3, cleaved caspase 3, cleaved caspase 8, BAD, phospho-Erk1/2 (Thr202/Tyr204; Cell Signaling), and β-tubulin (Sigma). Goat anti-mouse Ig-G-horseradish peroxidase (HRP), goat anti-mouse IgM-HRP, and goat anti-rabbit IgG-HRP were purchased from Pierce.

### Anti-angiogenesis assay

For our anti-angiogenic assays we used the murine MS1 microvascular endothelial cell line, selected due to its high VEGFR2 expression and responsiveness to VEGF. Inhibition of MS1 cell proliferation in the presence and absence of VEGF was evaluated using DNA incorporation of BrdU. MS-1 cells were serum starved overnight and the next morning preincubated with various concentrations of FC101 for 30 minutes. Next, 50 ng/ml VEGF or PBS alone (i.e., control) was added to respective wells along with BrdU and incubated for 4 hours. Cells were then fixed and BrdU incorporation measured by ELISA.

### In vivo tumorigenicity assay

All animal studies were carried out following the USDA, PHS, and NIH animal use and care guidelines. The animal protocol was approved by the Institutional Animal Care and Use Committee at the Louisiana State University Health Science Center in Shreveport (LSUHSC-S) (Protocol Number, P10-045). SCID Beige mice (CB17/Icr.Cg-Prkdc^scid^Lyst^bg^/Crl) were maintained on AIN76A semi-purified diets (Dyets, Bethlehem, PA). Mice were administered FC101 or PBS by IP injections 5 days per week. Total daily drug intake was 8 mg/kg. On day 3, groups of 8 mice (6–7 week old) were injected subcutaneously (s.c.) in the dorsal region with 1 × 10^6^ SRB12-p9 cells in 0.1 ml PBS. Tumors were measured five times weekly by digital caliper using the formula V = ((W + L)/4))^3^ × 4/3π, where L = length and W = width. Tumors were harvested and bisected at 25 days post-injection, or at the time of attaining a volume of 2 cm^3^. Tumors were fixed in formalin for histological analysis.

The differences between the groups of mice in terms of tumor volume or persisting benign cysts was compared using the Mann–Whitney test and differences in latency to tumor formation and survival were calculated by the Log-rank test and Kaplan-Meier Cumulative Survival tests.

### Statistical analysis

Statistical analyses were completed using *ANOVA*, followed by Fisher's protected least significant difference procedure. A p-value of < 0.05 (2-tail) was considered statistically significant.

## Abbreviations

FC101: Fusarochromanone
VEGF-A: Vascular endothelial growth factor-A.
